# Adjusting Potentially Confounded Scoring Protocols for Motivation Aggregation in Organismic Integration Theory: An Exemplification with the Relative Autonomy or Self-Determination Index

**DOI:** 10.3389/fpsyg.2016.00272

**Published:** 2016-02-29

**Authors:** Ali Ünlü

**Affiliations:** Centre for International Student Assessment (ZIB), Technical University of Munich (TUM)Munich, Germany

**Keywords:** self-determination theory, organismic integration theory, motivation internalization, confounded scoring protocol, adjusted relative autonomy index

## Introduction

Scoring protocols are summary statistics aggregating individual test or subscale scores to yield an overall informative measure. For the questionnaires used to assess the constructs postulated in the organismic integration sub-theory of self-determination motivation theory—in this sub-theory motivation is conceptualized as a continuum with polar and intermediate types of motivation that vary in the extent to which they are internal or external—, the scoring protocols are designed without taking into account the fact that intermediate motivation types may generally represent a mixture of internal as well as external motivation. Thus, in the process of weighting the subscale scores, the same weights are used in the protocols for the shares of internal and external motivation of a regulation type, thereby confounding the resulting overall measure, which therefore may lack interpretability. As a consequence, subsequent analyses based on such an aggregation measure may be distorted or erroneous. This can be important from a practical viewpoint, e.g., when investigating correlations of the measure with other more substantial variables of a theory or study.

In this article, I use an example of a scoring function, the RAI (or SDI) index, commonplace in many areas of motivation research, to exemplify how adaptations can be made to accommodate biasing effects on the overall index value that may result from the confounding of internal and external motivation. The approach can even be generalized and applied to other scoring protocols, which can be adjusted for mixed or confounded internal and external motivation in an analogous manner, as exemplified in this article with the RAI index. Thus, I advocate adjusting for such effects by proper choice of a scoring protocol formula and of the weights used for the motivation types combined therein.

## Self-determination theory

*Self-determination theory* was proposed by Deci and Ryan (1985, 2000, 2002) and is a popular theory of motivation. This theory is useful for understanding the motivational basis of human behaviors. The general aim is to investigate the interplay between the extrinsic forces or factors acting on people (e.g., grades, evaluations, or payment) and the intrinsic motives or needs inherent in humans (e.g., interests, curiosity, or enjoyment). Applications in substantial fields, such as in education, health care, or organizations, are numerous and are extensively referenced, with comprehensive additional materials on the theory and the available questionnaires, on the website www.selfdeterminationtheory.org.

**Figure 1:**
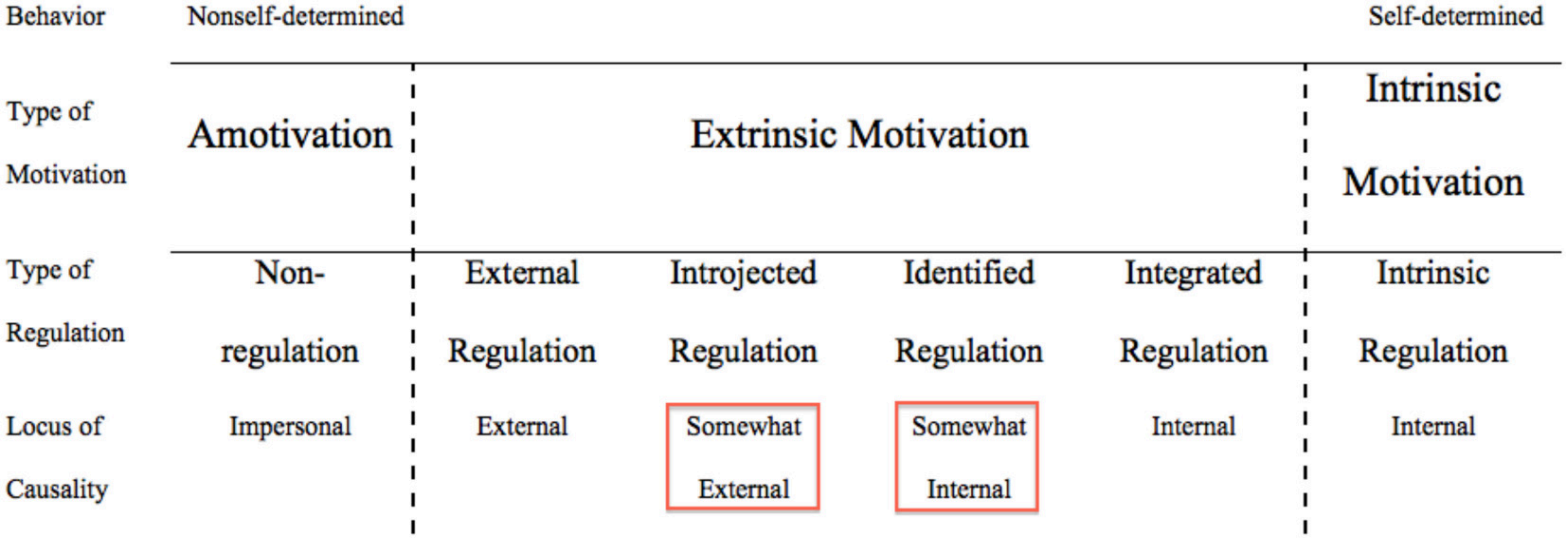
**The self-determination continuum is shown, including the intermediate types of identified regulation and introjected regulation of extrinsic motivation (Deci and Ryan, 2000)**.

Self-determination theory is composed of different mini-theories such as the *organismic integration theory*. With this sub-theory researchers particularly address extrinsic motivation in its various forms and distinguish between different regulation types of motivation. Why? An activity may start with being purely extrinsic at the outset; e.g., you may not be intrinsically motivated (not liking or enjoying) to do fractional arithmetic. However, while working on a fractions task, a person may value the activity more and more, and it may become part of one's self, thereby being gradually internalized. According to organismic integration theory behaviors can move from being purely extrinsic to being completely intrinsically motivated and these types of motivation regulation are ordered along a continuous scale called the *self-determination continuum* (see Figure 1).

Deci and Ryan (2000) segmented the continuum into three different motivation: intrinsic motivation, extrinsic motivation, and amotivation (not further considered). The four regulation types of extrinsic motivation are, in increasing order of internalization, external regulation, introjected regulation (*somewhat external*), identified regulation (*somewhat internal*), and integrated regulation.^1^ Intrinsic motivation is not further differentiated and referred to as intrinsic regulation. In self-determination theory researchers maintain that the more self-determined or internalized extrinsic motivation is, the deeper or better the observed behaviors or outcomes will be (e.g., Grolnick and Ryan, 1987; Vansteenkiste et al., 2005; Stefanou et al., 2013). At this point it is important to note that the intermediate types of identified regulation and introjected regulation of extrinsic motivation are assumed to lie in between the completely internal and completely external “extremes” or poles of intrinsic regulation and external regulation of the continuum, respectively (Figure 1), thereby leading to varying extent to which these intermediate types of motivation regulation are internal or external.^2^

## Analysis of motivation internalization or externalization

In Ünlü and Dettweiler (2015), a quantification approach was introduced and illustrated with empirical data on the science learning motivation of pupils in different pedagogical settings, which allowed gauging of the extent of internalization or externalization of the identified and introjected regulation types of extrinsic motivation. The question of interest was a constrained regression or least squares problem. For identified regulation *IdR*, for instance, the extent to which this regulation can be viewed as internal and external, here represented by the model parameters π_1, *IdR*_ and π_2, *IdR*_, respectively, were computed based on the convex decomposition model

$$IdR=\pi_{1,IdR}InR+\pi_{2,IdR}ExR,$$

where intrinsic regulation *InR* and external regulation *ExR* are the completely internal and completely external motivation poles of the theory, and the proportion weights π_1, *IdR*_ ≥ 0, π_2, *IdR*_ ≥ 0 with π_1, *IdR*_ + π_2, *IdR*_ = 1 are the parameters that were estimated from the data. Analogously, the extent to which the introjected regulation type *IjR* can be viewed as internal and external were computed in Ünlü and Dettweiler (2015), i.e., the weights π_1, *IjR*_ and π_2, *IjR*_, respectively. This yielded quantifications of the intermediate motivation internalizations that were vaguely expressed as *somewhat internal* and *somewhat external*, and which remained undetermined in the organismic integration sub-theory of self-determination theory.

I want to argue that these computable internalization or externalization shares could and should, be used in scoring protocols that include and weight, in their formulations, such intermediate motivation types as the identified regulation or introjected regulation, along a posited self-determination continuum. The following sample scoring protocol helps to illustrate the point.

## Adjusting the relative autonomy index for mixed or confounded internal and external motivation

A popular scoring protocol in self-determination theory is the *relative autonomy index* (RAI), also known as the *self-determination index* (SDI). For details, see Grolnick and Ryan (1989), Ryan and Connell (1989), Levesque et al. (2004), Vallerand (2007), and Kusurkar et al. (2013). The reader can see the pertinent works by Wilson et al. (2012) and Chemolli and Gagné (2014) for critique on this index and for other approaches to instrument scoring, such as the bifurcation scoring protocol (Wilson et al., 2012), which can be adjusted for mixed or confounded internal and external motivation in an analogous manner, as exemplified in this article with the RAI index.

With the RAI (or SDI) index, subsequently in its version for instruments assessing extrinsic motivation and intrinsic motivation, the inventory scores are weighted and combined to give a descriptive overall measure of the behavioral self-regulatory style. The formula is

RAI≡SDI=(2InR+IdR)−(2ExR+IjR)​.

I propose an adjusted variant of this scoring rule. The regulation types *IdR* and *IjR* vary in the extent to which they are internal and external, and the goal is to disentangle these and weight them separately. An *adjusted* RAI (or SDI) index can be defined as

$$\begin{array}{l} {\text{RAI}}_{\text{adj}}\equiv {\text{SDI}}_{\text{adj}}\\ \;=\text{​}mean\;internal\;motivation-mean\;external\;motivation, \end{array}$$

where *mean internal motivation* and *mean external motivation*, i.e., $\bar{\text{IM}}$ and $\bar{\text{EM}}$, respectively, can be quantified based on the internalization or externalization shares, i.e., using the π-weights described above, computed according to the method discussed in Ünlü and Dettweiler (2015):

$$\bar{\text{IM}}=\frac{\left(InR-1\right)+\pi_{1,IdR}\left(IdR-1\right)+\pi_{1,IjR}\left(IjR-1\right)}{3}$$

and

$$\bar{\text{EM}}=\frac{\left(ExR-1\right)+\pi_{2,IdR}\left(IdR-1\right)+\pi_{2,IjR}\left(IjR-1\right)}{3}.$$

Translation with −1 and averaging are applied to ensure that in the instrument variables *InR* − 1, *IdR* − 1, *IjR* − 1, *ExR* − 1 and the new scoring protocol RAI_adj_ ≡ SDI_adj_, all of these variables range in the same interval, from 0 to, e.g., 4 (cf. Müller et al., 2007), i.e., from lowest to highest motivation scores. This is not the case for the original RAI index. In both variants, larger values of the protocols imply more internalized or self-regulated motivational behavior.

The original RAI index does not allow one to account for the extent to which the identified and introjected regulation types are internal and external. In the process of weighting the subscale scores, the same weights are used (1 or −1, respectively). In contrast, the alternative adjusted RAI is weighted according to the extent to which these regulation types are internal and external. For example, π_1, *IdR*_(*IdR* − 1) represents the amount of internal motivation of identified regulation, and π_2, *IdR*_(*IdR* − 1) is the amount of external motivation. In sum, these mixture components do yield the overall variable identified regulation, i.e., π_1, *IdR*_(*IdR* − 1) + π_2, *IdR*_(*IdR* − 1) = *IdR* − 1.

Therefore, I conclude the traditional RAI scoring rule may generally lack interpretability. I suggest that, as a minimum requirement, the current RAI scoring rule, when used, should be compared to some adjusted variant of it (such as the introduced variant), and if possible, be studied in combination with other scoring rules as well.

## Conclusion

In self-determination motivation research, aggregated score statistics, in particular the RAI coefficient, have been used in theoretically substantial analyses as a substitute for the motivation inherent in a participant. For example, scoring protocols can be correlated with essential variables of a theory or study. It remains to be seen how robust or sensitive the derived fundamental statements or interpretations based on possibly confounded scoring protocols are, when adjusting or correcting adaptations are utilized in the manner I proposed with this article. Therefore, previous motivation studies could be reanalyzed, based on adjusted scoring protocol variants, aiming at replicating results or deriving similar or new findings. Future research into this issue is obviously needed, including systematic comparisons through in-depth real data applications. I think that this topic is an interesting and important one to pursue in future motivation research, particularly, to analyze, in light of these findings, previously published application studies on motivation.

## Author contributions

The author confirms being the sole contributor of this work and approved it for publication.

### Conflict of interest statement

The author declares that the research was conducted in the absence of any commercial or financial relationships that could be construed as a potential conflict of interest.

## Footnotes

^1^Introjected and identified regulations are the motivational styles that are directly relevant to this article. Introjected regulation means a person is acting on the basis of external social or societal expectations that were only partially internalized and remain somewhat external to the self. Identified regulation can be observed if the person has identified with the external values of their behaviors and has internalized these more into her/his own value system. For details, see Deci and Ryan (1985, 2000, 2002).

^2^This geometric view is central to the convex quantification approach reviewed below and methodologically well-defined as the regression weights within the approach based on the motivational regulation types measured using the available questionnaires in self-determination theory. According to this decomposition model, the parameters are interpreted to quantify the extent of motivation internalization or externalization pertaining to the notion of *somewhat internal* and *somewhat external* on the self-determination theory subscales of identified regulation and introjected regulation, respectively.
